# A Novel Simple Approach to Material Parameters from Commonly Accessible Rheometer Data

**DOI:** 10.3390/polym12061276

**Published:** 2020-06-03

**Authors:** S. Schrüfer, D. Sonnleitner, G. Lang, D. W. Schubert

**Affiliations:** 1 Department of Materials Science and Engineering, Institute of Polymer Materials, University Erlangen-Nuremberg, Martensstraße 7, 91058 Erlangen, Germany; stefan.schruefer@fau.de; 2 Bavarian Polymer Institute, Key Lab Advanced Fiber Technology, Dr.-Mack-Straße 77, 90762 Fürth, Germany; 3 Research Group Biopolymer Processing, University of Bayreuth, Ludwig-Thoma-Straße 36A, 95447 Bayreuth, Germany; david.sonnleitner@uni-bayreuth.de (D.S.); Gregor.Lang@uni-bayreuth.de (G.L.)

**Keywords:** rheology, rheological model systems, parameter determination

## Abstract

When characterizing the viscoelastic properties of polymers, shear rheological measurements are commonly the method of choice. These properties are known to affect extrusion and nozzle-based processes such as fiber melt spinning, cast film extrusion and 3D-printing. However, an adequate characterization of shear thinning polymers can be challenging and still insufficient to not only describe but predict process relevant influences. Furthermore, the evaluation of rheological model systems in literature is mostly based on stress–relaxation experiments, which are rarely available for various polymeric materials. Therefore, a simple approach is presented, that can be used to evaluate and benchmark a wide range of rheological model systems based on commonly accessible frequency sweep data. The approach is validated by analyzing alginate PH176 solutions of various concentrations, a thermoplastic poly-urethane (TPU) Elastollan 1180A melt, the liquid silicon rubber Elastosil 7670 and a polycaprolactone (PCL) fiber-alginate composite system. The used rheological model systems, consisting of simple springs and dashpots, are suitable for the description of complex, viscoelastic material properties that can be observed for polymer solutions and gel-like systems. After revealing a suitable model system for describing those material properties, the determination and evaluation of relevant model parameters can take place. We present a detailed guideline for the systematic parameter revelation using alginate solutions of different concentrations as example. Furthermore, a starting point for future correlations of strut spreading in 3D-bioprinting and model parameters is revealed. This work establishes the basis for a better understanding and potential predictability of key parameters for various fabrication techniques.

## 1. Introduction

The prediction of material behavior can lead to immense savings, monetary and time wise, in various industrial processes. A commonly chosen method for such predictions is the analysis of rheological material properties. The subsequent modelling of generated data can potentially further expand its predictability exceeding the underlying experimental range. Due to the advances in computational engineering and technology, many researches focus on constitutive modeling, based on linear and nonlinear continuum mechanics [1,2] and computational modeling using a variety of finite element approaches [3,4]. Computational simulations are often highly specific due to the chosen boundary conditions, while continuum mechanical approaches require a deep knowledge of the underlying physical and mathematical tools. A more accessible approach for rheological modelling is based on classic mechanics following Hooks law (springs) and Newtonian behavior (dashpot). Examples of this approach can be found in the literature since the 1960s [5], where already correlations of rheological properties and asphalt mixtures have been investigated. Since this time, the application of rheological model systems has continued, covering a wide range of industrial related research fields. Dey et al. [6] used the Burgers model parameters, derived from stress relaxation tests for further characterizing the response of soil beds to viscoelastic loads. He concluded the Burgers model as suitable for describing a majority of occurring time dependent visco-elastic effects and explicitly suggests its use for geotechnical engineering. Mazurek et al. [7] modelled the stiffness of asphalt concrete within the linear viscoelastic regime. He also compared the applicability of different higher order model systems, such as Burgers model, Huet–Sayegh, and generalized Maxwell model and concluded the Huet–Sayegh model was the most suitable one. While they deemed the Burgers model as not suitable for modelling the asphalt concrete behavior, they also included a table that shows an adequate R^2^ and mean normalized error while fitting the obtained complex modulus data. Xu et al. [8] took this approach even further and used mathematical functions, derived from rheological models, in order to generate a master curve for the measured dynamic moduli. Mahiuddin et al. [9] displayed a completely different use for rheological modelling. He dedicated his research to the characterization of mechanical properties of fruits and vegetables in order to optimize transportation, storage and handling of harvested goods. Therefore, he compared the applicability of three elements generalized Maxwell model with a fractional power-law model, solved using Caputos’ fractional derivate. By using the fractional power-law model he could reduce the amount of fit parameters from seven to four, while still guaranteeing an adequate description of the measured stress–relaxation data. In the eyes of the authors, a reevaluation of the showcased data using the Burgers model, which uses four fitting parameters as well, should yield an adequate description as well. Shi et al. [10] and Meng et al. [11] used the three and four parameter Burgers model to predict stress relaxation properties of starch films and rice flour. An accurate description of the stress–strain relation and creep properties was achieved. Kundera et al. used a generalized Maxwell approach to describe the visco-elastic stress recovery behavior of O-rings, produced by an additive manufacturing approach [12]. A generalized Maxwell model, using five fitting parameters, was used to describe the stress–relaxation properties of the sealings. However, only the mechanical properties were correlated with the rheological model parameters. A direct correlation of sealing properties and model parameters did not take place. The here shown examples are representative for a wide field of rheological modelling. The majority of modelling approaches is based on the stress–relaxation test procedure [13]. While this is of major interest for the characterization of product properties, a lot of information affecting the production process is neglected. For nozzle and extrusion based processes, which play a major role in polymer processing and its correlated industrial fields, an analysis of frequency depended material properties is mandatory. However, research covering the classic rheological modelling of frequency dependent measurements is rarely conducted. Bhattacharyya et al. [14] derived a predictive function from an augmented Jeffreys model, where a friction element is added, for the dampening properties of shape memory polymers. He also derived such functions for various mechanical loading types, covering constant stress and stress rate as well as constant and periodic strain and constant strain rate. Sun et al. [15] used the Burgers model in a similar way to the presented approach. After a rheological characterization via frequency sweep, two fit functions for G’ and G″ were derived and fitted to the resulting data. However, they concluded that the Burgers model is not suitable for describing frequency dependent measurement data of dough. Our own experience shows that fit functions, as used in the publication, can lead to falsified conclusions due to the use of too many fit parameters. The data presented was subsequently fitted by a two parameter power-law function. However, shear rate or frequency dependent properties were not described by rheological model systems.

The here presented approach is meant to close the gap between commonly performed rheological measurements for polymer characterization and rheological modelling. We based the here presented method on frequency sweep measurements, where the material is subjected to an oscillatory strain with fixed amplitude and varied frequency. Due to the well-known phenomena during such measurements it is easy to find pertinent literature for benchmarking own findings. Additionally, the amount of data that is already openly accessible enables wide-ranging conclusions, exceeding the scope of self-performed experiments. In order to proof the applicability of our approach we investigated different types of common polymer materials. Uncrosslinked alginate solutions with various concentrations are chosen as representative for rheological liquid behavior [16]. Due to their properties, alginates are used for a variety of applications, which range from seemingly simple products, such as thickeners for the food industry [17], to complex drug delivery systems [18] and wound dressings [19]. The ability to store water, transport important components for cell proliferation, such as nutrients and growth factors, and to provide a mechanically stable surrounding for cell transport also makes alginate usable as bio-ink for 3D-printing processes [20,21]. An emerging research field, namely biofabrication, uses alginates and comparable hydrogels for the fabrication of tissue and organ replacement. Due to the connection of the 3D printing process and the rheological properties of bioinks one can find a variety of rheological characterization protocols and their results [22,23,24,25]. Additionally, one can potentially reevaluate studies such as Gao et al. [26], which correlates frequency dependent material properties and the resulting printing quality for better comparability of various printed materials. A possible approach on the correlation of rheological model parameters and bio-printing of alginate is presented, as follows.

As example for polymer melts we chose a thermoplastic poly-urethane (TPU). This thermoplastic material type can be used for the fabrication of elastic products in typical polymer processing approaches such as melt-spinning [27], 3D-printing [28,29] and the production of flexible sensors [30]. Here, rheological modelling can potentially yield further insights on macromolecular processes, which are directly connected to the frequency dependent properties.

For the sake of completeness, we furthermore characterized a liquid silicone rubber system (LSR). This highly crosslinked rubber is chosen as representative for rheological solids. Such LSR materials are commonly combined with conductive filler materials, often carbon particles and carbon nanotubes, in order to generate sensor systems [31,32,33] or artificial actuators for a variety of applications [34,35,36]. However, a correlation of rheological measurements and process or product performance is rarely conducted. Thus, an approach to further evaluate rheological measurements is presented. This method can be used to determine a suitable rheological model system as well as revealing model parameter values. As highlighted before, a variety of materials has already been analyzed with respect to their creep recovery behavior. However, to the knowledge of the authors, the only description of frequency dependent material behavior is based on the derivation of fit-functions from rheological model systems, which can be error-prone and inapplicable. Therefore, the novelty of the presented work can be concluded in three main aspects:

(1) A novel, yet simple, concept is suggested to directly reveal the constitutive equation of a material from rheological measurements. In particular, the constitutive equation in the form of a differential equation is directly accessed, relating stress σ and deformation ε.
(2) Various rheological model systems are benchmarked to reveal the simplest and statistically most significant ones. The presented approach is applicable to Dynamic Mechanical Analysis (DMA; typically used symbols are σ for stress and ε for deformation) and shear rheology (τ for stress and γ for deformation).
(3) We present a material independent approach that is suitable for further in-depth interpretation of frequency dependent material properties, known to be of major importance for a variety of industrial applications. The presented concept additionally allows the analysis of specific model parameters and their respective influences on the targeted applications. Hypothetically, one can correlate key processing parameters such as shape fidelity for additive manufacturing or sealing properties for rubbers to the resulting model parameters. It should be noted that huge amounts of data are required in order to reveal correlations between model and process parameters. This paper, however, will focus on the approach of generating necessary data for possible future correlations.

## 2. Materials and Methods

### 2.1. Theory/Strategy

The typical output from a rheometer is G’ and G″, the storage and loss modulus respectively. Running a so called frequency-sweep one obtains G’(ω) and G″(ω) as a function of angular frequency ω, or in a DMA E’(ω) and E″(ω), where the angular frequency ω typically covers 2 to 3 orders of magnitude.

For oscillatory measurements the applied deformation can be written as:

(1) $$\varepsilon \left(t\right)=\tilde{\varepsilon }\cdot \sin\left(\omega t\right)$$

where $\tilde{\varepsilon }$ is the known deformation amplitude in a strain controlled rheometer [37].

The corresponding stress can be calculated utilizing the output from the rheometer or the DMA with:

(2) $$\sigma \left(t\right)=E^{\prime }\left(\omega \right)\cdot \tilde{\varepsilon }\cdot \sin\left(\omega t\right)+E^{\prime \prime }\left(\omega \right)\cdot \tilde{\varepsilon }\cdot \cos\left(\omega t\right)$$

From Equations (1) and (2) the higher derivatives with respect to time are also accessible for ε(t) and σ(t):

(3) $$\dot{\varepsilon }\left(t\right)=\tilde{\varepsilon }\cdot \omega \cdot \cos\left(\omega t\right)$$

(4) $$\ddot{\varepsilon }\left(t\right)=-\tilde{\varepsilon }\cdot \omega^{2}\cdot \sin\left(\omega t\right)=-\omega^{2}\cdot \varepsilon \left(t\right)$$

(5) $$\dot{\sigma }\left(t\right)=E^{\prime }\left(\omega \right)\cdot \tilde{\varepsilon }\cdot \omega \cdot \cos\left(\omega t\right)-E^{\prime \prime }\left(\omega \right)\cdot \tilde{\varepsilon }\cdot \omega \cdot \sin\left(\omega t\right)$$

(6) $$\ddot{\sigma }\left(t\right)=-E^{\prime }\left(\omega \right)\cdot \tilde{\varepsilon }\cdot \omega^{2}\cdot \sin\left(\omega t\right)-E^{\prime \prime }\left(\omega \right)\cdot \tilde{\varepsilon }\cdot \omega^{2}\cdot \cos\left(\omega t\right)=-\omega^{2}\cdot \sigma \left(t\right)$$

Due to the fact that the Equations (1)–(6) are periodic functions, sampling over one period is sufficient to generate corresponding data in $\varepsilon \left(t_{i}\right)$, $\dot{\varepsilon }\left(t_{i}\right)$, $\ddot{\varepsilon }\left(t_{i}\right)$, $\sigma \left(t_{i}\right)$, $\dot{\sigma }\left(t_{i}\right)$, and $\ddot{\sigma }\left(t_{i}\right)$, where i is an index considering the sampling over one period. A sampling over 10 time steps t_i_ is sufficient, and can be chosen arbitrarily. However, this must be done for each applied angular frequency $\omega_{j}$. Thus, t_ij_ corresponds to:

$$t_{\mathrm{ij}}=i\cdot \frac{2\pi }{\omega_{j}}\cdot \frac{1}{10}$$

with i ranging from 0 to 9, while j only indicates here the specific angular frequency $\omega_{j}$.

Therefore, a table with the following structure is available (see Table 1):

On the basis of the calculated data, one can evaluate step by step various rheological model systems by modeling σ as a function of ε, $\dot{\varepsilon }$, $\ddot{\varepsilon }$, $\dot{\sigma }$, and $\ddot{\sigma }$, where not always all contribution as a so called main effect are needed. For better understanding, the most common rheological model systems are discussed briefly in the experimental and model evaluation section.

From Table 2 it is evident that the Maxwell model is the suitable model if a linear equation can describe the stress σ as a function of $\dot{\varepsilon }$ and $\dot{\sigma }$. If the stress can be better described by a linear function of ε and $\dot{\varepsilon }$, the Kelvin–Voigt model should be applied.

Further relevant models are shown in Table 2 and Table 3 and can thus be evaluated by describing the stress σ according to the linear equations given in the respective columns [38]. One can also deduce from Table 2 and Table 3 that both variations of the Zener model as well as Lethersich and Jeffreys model systems are indistinguishable due to the structure of their differential equations.

It must be pointed out that, e.g., for shear, models like the Burgers model or even simpler, the Maxwell model are consistent with a shear thinning behavior if only the viscosity is considered. The complex viscosity $\eta^{*}$ relates applied shear rate $\dot{\gamma }$ to shear stress τ, while the complex shear modulus $G^{*}$ relates applied shear γ to shear stress τ in the oscillatory rheological experiment:

$$\tau =G^{*}\cdot \gamma =\left(G^{\prime }+{\mathrm{iG}}^{\prime \prime }\right)\gamma \;\tau =\eta^{*}\cdot \dot{\gamma }$$

From the Cox–Merz rule the shear rate dependent viscosity follows with:

$$\eta \left(\dot{\gamma }\right)=\left|\eta^{*}\left(\omega =\dot{\gamma }\right)\right|=\frac{\sqrt{G^{\prime 2}+G^{\prime \prime 2}}}{\omega }$$

The reason for determining the parameters $K_{i}$ is to reveal the individual material parameters $E_{i}$ and $\eta_{i}$ by solving the system of equations, which result from the comparison of coefficients between linear equations and σ−forms of the differential equation. K parameters can be accessed by using a linear regression model, correlating occurring stress σ and the relevant main effects (obtainable from Table 2 and Table 3) in statistic software packages such as Statistica, SPSS or Excel. Furthermore, the theoretical stress, also titled calculated stress, determined by the linear regression can be compared to real stresses, measured by the rheometer. As an example, using the Burgers model leads to the following relations:

(7) $$K_{1}=\eta_{1}$$

(8) $$K_{2}=\frac{\eta_{1}\eta_{2}}{E_{2}}$$

(9) $$K_{3}=-\left(\frac{E_{1}\eta_{1}+E_{1}\eta_{2}+E_{2}\eta_{1}}{E_{1}E_{2}}\right)$$

(10) $$K_{4}=-\frac{\eta_{1}\eta_{2}}{E_{1}E_{2}}$$

Solving the resulting system of equations, one can calculate the individual model parameters for the burgers system as follows:

(11) $$E_{1}=-\frac{K_{2}}{K_{4}}$$

(12) $$E_{2}=\frac{E_{1}K_{1}}{-K_{3}E_{1}-\frac{E_{1}K_{2}}{K_{1}}-K_{1}}$$

(13) $$\eta_{1}=K_{1}$$

(14) $$\eta_{2}=\frac{K_{2}E_{2}}{K_{1}}$$

Rheological models are already used to describe the stress–relaxation properties of different material systems such as rubbers for O-rings [12]. The Burgers model itself has already been demonstrated to be a suitable system for the description of visco-elastic properties of hydrocolloidal systems such as flour dough [15], rice flour pasting [11], and methoxyl pectin gels [39]. However, those evaluations are either based on stress–relaxation experiments or on error prone fitting of experimental data. The approach presented here is both easy to use and performable on existing shear rheological data, which is widely accessible in the literature. Hypothetically, the resulting parameters hold information about the structural integrity of hydrogel struts after 3D-printing. A further correlation of model parameters and printing results will be the focus of further research.

### 2.2. Sample Preparation for Rheological Characterization

In this work, several solutions of sodium alginate PH176 (VIVAPHARM^®^, J. Rettenmaier & Söhne GmbH & Co KG, Rosenberg, Germany) were used. The concentration is varied from 2% (*w/v*) to 8% (*w/v*). Sample preparation was performed according to [40]. The storage time at 37 °C was reduced to 16 h, since complete solubility had already been achieved. This holds true for sample concentrations up to 5%. Alginate solutions with higher polymer content were stored for 24 h before measurements took place in order to ensure homogenous solution properties. Constant stirring was performed with a magnetic stirrer at 150 rpm. However, stirring was not possible for concentrations higher than 4% due to the high viscosity of the solution. A calcium and magnesia free Dulbecco’s phosphate buffered saline solution DPBS (used as purchased; Sigma Aldrich, St. Louis, MO, USA) was used as solvent in order to prevent uncontrolled ionic crosslinking of the alginate solutions. For the fiber composite samples, PCL fiber fragments are added as dry filler in total weight percent simultaneously with the alginate powder. The stirring time of 16 h was also sufficient for a homogenous filler distribution.

The used liquid silicon rubber Elastosil 7670 (Wacker Chemie AG, Germany) was mixed in a 1:1 ratio (A:B) using a SpeedMixer DAC 150 SP (Hauschild Engineering, Hamm, Germany) with 1000 rpm for 1 min and 2000 rpm for 2 min, subsequently. The used coated steel mold was placed in a desiccator under vacuum in order to remove possible air inclusions. To ensure complete crosslinking the molds were rested for 18 h at room temperature.

As TPU an Elastollan 1180 A (BASF AG, Ludwigshafen, Germany) was used. Before rheological characterization could take place, disks of 25 mm diameter and approximately 2 mm height were pressed. The material was melted for 7 min at 210 °C and 10 bar pressure using a vacuum press. Afterwards, a pressure of 100 bar was applied for 3 min to ensure bubble free samples. Vacuum was applied during the whole preparation process in order to minimalize polymer degradation.

### 2.3. Shear Rheology

For alginate, two different rheometers, DHR-3 and ARG-2 (TA Instruments, New Castle, DE, USA), are used with a cone-plate geometry (Diameter 40 mm, 2° angle, 65 µm gap).

Preliminary tests proved both rheometers to yield identical results. All measurements are performed at 25 °C, regulated using a Peltier-element. A solvent trap with an additional liquid reservoir is used to prevent drying of the samples during measurements. Both reservoirs are filled with the same DPBS solution as used for the sample preparation. An optical evaluation of the gap filling showed no significant changes during the tests, hence effects of drying on the measurement results can be neglected.

For the determination of the linear viscoelastic regime an amplitude sweep is performed at moderate frequencies. An angular velocity of 10 rad/s is chosen arbitrarily for all samples. The amplitude used for the following frequency sweeps is determined by the influence of noise for very small amplitudes and by a sufficient distance from the critical amplitude in order to prevent non-linear material behavior or artifacts due to wall-slip [41]. Frequency sweeps range from 0.1 rad/s to 100 rad/s, since higher frequency measurements, especially for low concentration, are often erroneous due to the machine inertia. The frequency regime, however, can be adjusted to the region of interest for the specific application. Furthermore, the frequency sweep should cover at least three decades of frequencies to guarantee reliable data, for a subsequent analysis as shown in “Section 2.1”.

For the translation of oscillatory measurements to real 3D-printing processes, flow measurements are additionally performed. The same measurement set-up is used, while the rotational speed is varied from 0.01 s^−1^ to 100 s^−1^ (revolutions per second). An optical evaluation of the gap after every measurement showed no negative influences, like drying or ejection of material during both measurements. The relation of Cox–Merz could be confirmed for the used alginate solutions [42].

The crosslinked liquid silicon rubber samples are measured analogous to alginate samples. However, a 20 mm plate-plate geometry is used. An axial compression event, applying 0.5 N force for 1 min, is added prior to the measurement, ensuring contact between samples and measurement geometry.

Elastollan 1180A is characterized comparably using a 25 mm plate-plate geometry at 210 °C. In order to prevent polymer degradation during the measurement nitrogen is used as ambient inert gas. Due to wall-slip phenomena, no flow sweep measurements are performed for the LSR and TPU samples.

According to Mezger [37], one can neglect the inhomogeneity within the stress field of a plate-plate measurement system for small amplitude oscillatory measurements, relative to the end of the linear viscoelastic regime. For the comparison of data sets generated with different measurement set-ups (plate-plate, cone-plate) a correction for the occurring stresses and resulting viscosities according to Pahl et al. [43] or Münstedt et al. [44] can be performed. Therefore, our method can be applied to data sets obtained by both plate-plate and cone-plate measurement. In particular, the correction of data obtained by plate-plate geometry is trivial and needs only plate diameter and gap distance if the constitutive material model follows one of the linear differential equations given in Table 2 and Table 3.

### 2.4. Printing of Alginate

Alginate solutions are printed using a self-built bio-printing system, based on a conventional three axes printer equipped with a non-heated glass printing bed. Firstly, alginate is filled into a 10 mL cartridge (Nordson, Westlake, OH, USA) and mounted on the self-designed printhead. The throughput is adjusted by regulating the applied pressure. A throughput of 0.12 g/s is chosen based on a fiber formation study according to [22]. No die swell was observed. An adequate printing speed was experimentally determined and fixed at 60 mm/s for all concentrations. Therefore, a constant ratio of printing speed and throughput is ensured. All printing experiments are conducted using a 20 G needle (Cellink, Boston, MA, USA) at room temperature (22–24 °C).

## 3. Results and Discussion

### 3.1. Rheological Model Evaluation of Alginate

The following section will present the evaluated rheological systems, starting from simple single parameter models, namely a spring for pure elastic behavior and a dashpot as pure viscos system, heading towards multiparametric models, including Lethersich, Jeffreys and Burgers models. As representatives, only the results for 2%, 5%, and 8% alginate solutions are shown here. For benchmarking the model systems the observed (or measured) values are plotted against the statistically predicted ones. In this work, we are using the calculated shear stress, given by statistica, against the measured shear stress, determined by the rheometer. The range of both values, however, depends on the used strain for the specific measurement. The function y = x, (a linear graph with the slope of 1 and intersection at 0) represents a hypothetically perfect description of the measured data by the evaluated model [45]. For a more precise evaluation, commonly used statistical parameters such as the correlation coefficient R, R^2^, and the adjusted R^2^ can be used.

#### 3.1.1. Single Parameter Model: Spring and Dashpot

**Figure 1:**
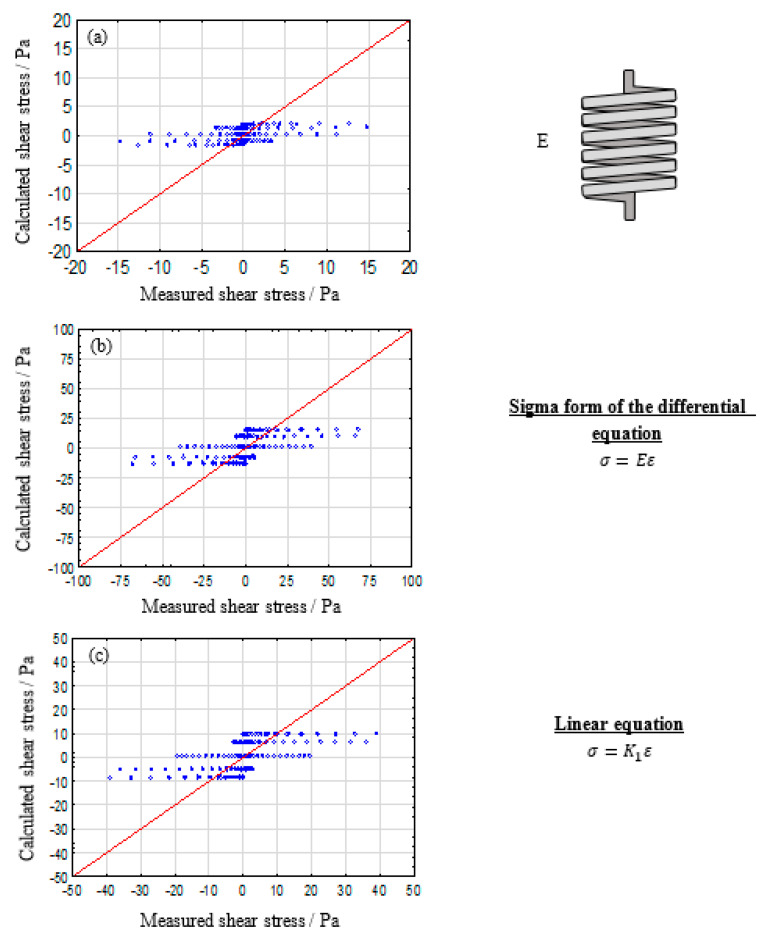
Calculated vs. measured shear stresses plot for a simple spring for describing the viscoelastic properties of 2% (**a**), 5% (**b**), and 8% (**c**) alginate solutions.

Figure 1 displays the evaluation of a single spring for describing the rheological behavior of alginate solutions. One can conclude that a single spring is not suitable for describing the measured material answer. If one takes the equation for a spring into consideration, no dependency on frequency is considered. Additionally, a spring-like material answer is, by definition, instantaneous and totally reversible. Therefore, the observed shear thinning and flow behavior cannot be described adequately.

**Figure 2:**
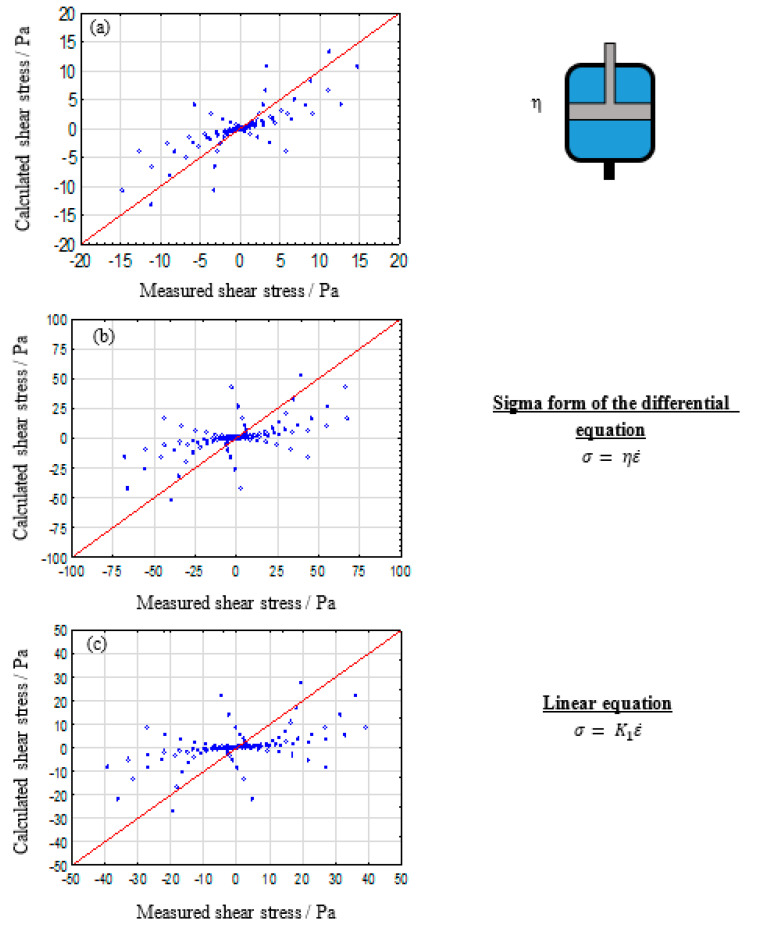
Calculated vs. measured shear stresses plot for a single dashpot for describing the viscoelastic properties of 2% (**a**), 5% (**b**), and 8% (**c**) alginate solutions.

The simplest model system that can describe strain rate and, in oscillatory measurements, frequency dependent material behavior, is represented by the dashpot. The results are shown in Figure 2.

It is evident that the single dashpot system is not suitable for a sufficient description of the viscoelastic properties of alginate solutions either. Particularly, higher concentrations show significant differences to the reference graph. However, for low alginate concentrations the pure dashpot depicts the desired trend. These observations can be clearly related to the properties of the components used. The DPBS puffer rheologically behaves like a Newtonian fluid (e.g., pure water), which causes the low concentrated alginate solutions to show more dashpot like behavior. However, the increasing polymer content also induces elastic properties in the system. Therefore, it can be concluded that alginate solutions neither show pure elastic nor pure viscos behavior, necessitating model augmentations towards multiparametric systems.

#### 3.1.2. Two Component Model Systems: Kelvin–Voigt and Maxwell

**Figure 3:**
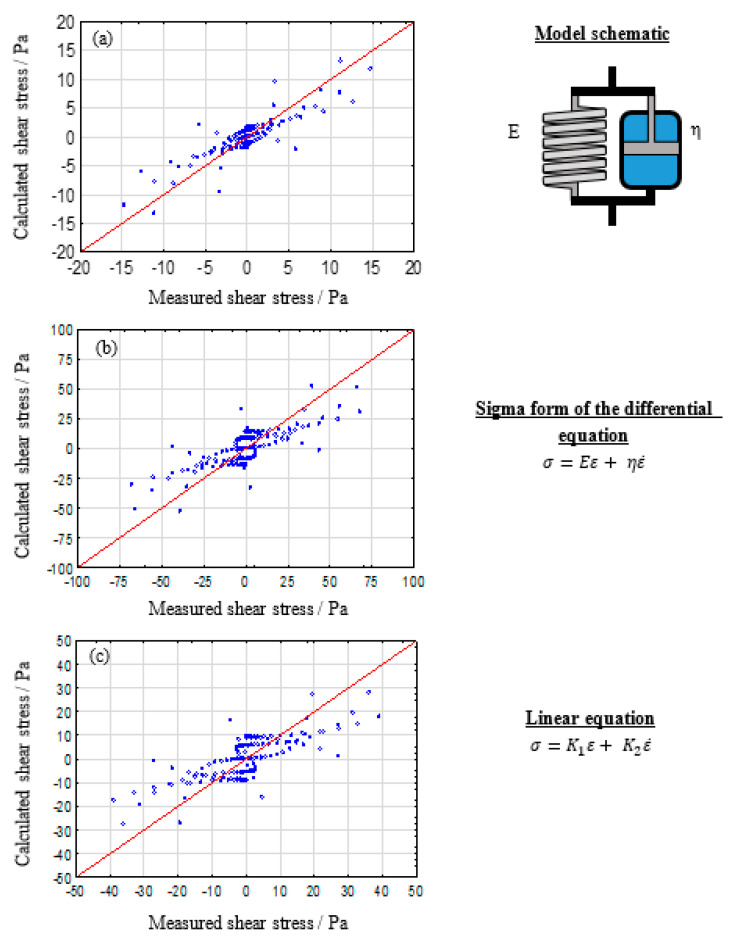
Evaluation of the Kelvin–Voigt model as suitable model to describe the viscoelastic properties of 2% (**a**), 5% (**b**), and 8% (**c**) alginate solutions.

The Kelvin–Voigt model, which consists of a parallel arrangement of one spring and one dashpot, is often referred to as suitable for describing viscoelastic solid behavior [37]. This behavior is defined by a time dependent deformation when a constant stress is applied, followed by total recovery when the applied stress disappears. Figure 3 shows the resulting observed vs. predicted plots. Even though a sufficient description of the material behavior is still not possible, a clear improvement in comparison to the single parameter models can be observed. It should be stated that the Kelvin–Voigt model can be theoretically suitable for highly crosslinked hydrogels. However, alginate solutions show a non-reversible rest deformation in stress–relaxation tests [46,47], which cannot be described by the Kelvin–Voigt model.

**Figure 4:**
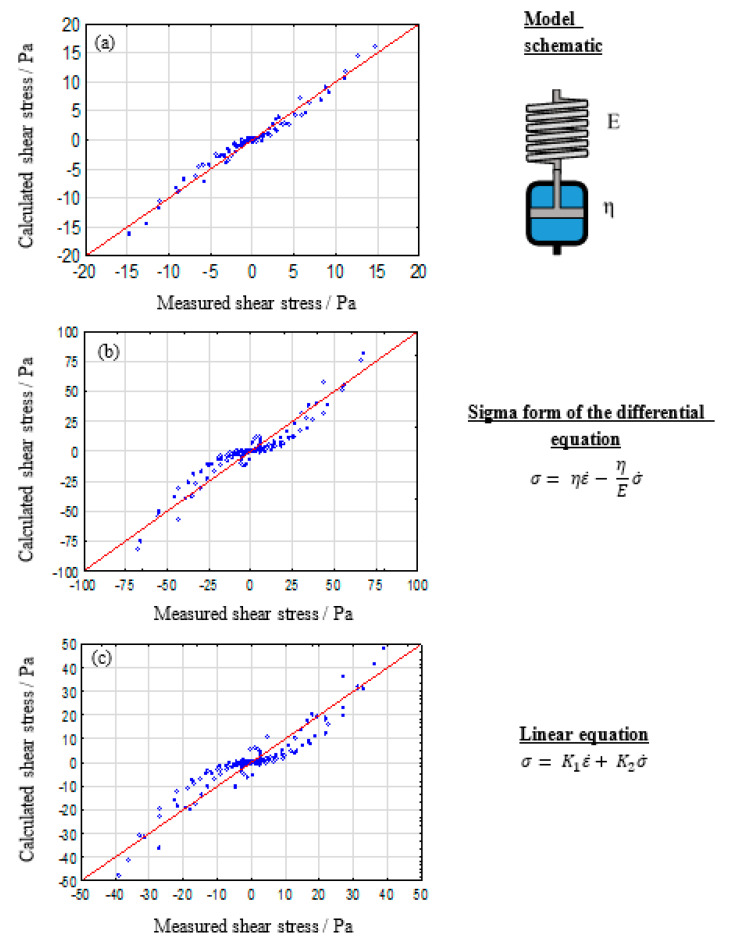
Calculated against measured shear stress plot for the evaluated Maxwell model for 2% (**a**), 5% (**b**), and 8% (**c**) alginate solutions.

The simplest model to describe viscoelastic liquid like behavior with non-recoverable deformation is the Maxwell-model, which consists of one spring and one dashpot that are arranged in series. The Maxwell evaluations are shown in Figure 4.

For low alginate concentration a good description of the measured data is possible. However, the quality of the model decreases for increasing polymer concentrations. This hints at the development of additional interactions between polymer macromolecules, which induce additional elastic properties that limit the non-recoverable flow behavior of the solutions. Since alginates can be described as block-co-polymers [48] a micro phase separation for high polymer concentration is conceivable. This hypothesis will be subject to future research in order to reveal detailed macroscopic models. Also, concentration differences, dividing the hydrogel into polymer-rich and polymer-poor regions, can influence the local elastic properties. It can be concluded that the Maxwell-model is suitable for describing low concentrated alginate solutions. Nevertheless, further model augmentations are necessary to reveal a wide-ranging model for the description of the viscoelastic properties of biofabrication relevant bioinks.

#### 3.1.3. Four Parameter Model: The Burgers Model

The Burgers model consists of a Maxwell and a Kelvin–Voigt model in linear arrangement.

**Figure 5:**
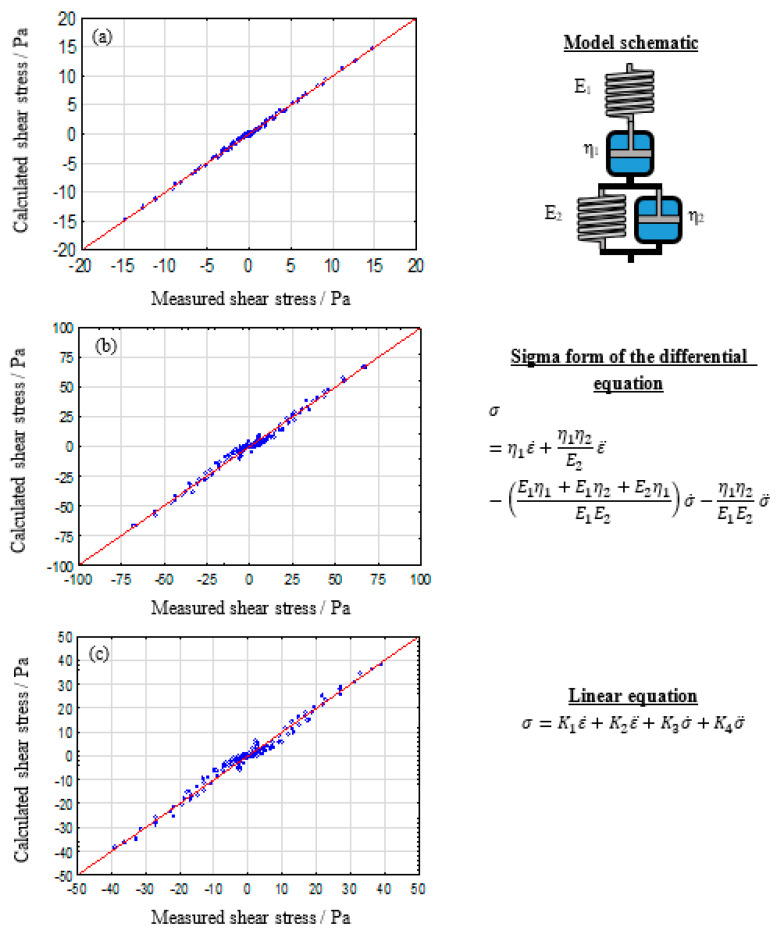
Calculated against measured shear stress plot for the used burgers model system for 2% (**a**), 5% (**b**), and 8% (**c**) alginate solutions.

Chirila et al. [49] analyzed the shear creep and recovery behavior and discussed the applicability of rheological model systems for various crosslinked poly(1-vinyl-2pyrrolidinone) hydrogels. The majority of tested gels could be well described by the Burgers model. Kocen et al. [50] described the creep recovery behavior of gellan gum mixed with bioactive glass particles using the burgers model, as well, hinting towards a good applicability of the burgers model for hydrocolloidal gels. Both publications also provide oscillatory frequency sweep data which were, however, not further investigated in hindsight of rheological model parameters, resulting in a potential information loss. The evaluation of alginate solutions based on shear rheological measurements is shown in Figure 5.

Figure 5 shows a nearly perfect description for low concentrated alginate solutions using the burgers model. Medium concentrated samples also show very good agreement with the reference graph. The systematic error, which is observable for high polymer concentration in other models, almost vanishes here. The clustering of measurement points close to (0/0) is still present. However, this is mainly caused by the use of a full oscillation of a sinus function for all calculations, which results in three zero values per full period. Overall, it can be concluded that the Burgers model is the most suitable system for a wide range of alginate solution concentrations. Therefore, the parameter calculations, described in the strategy section, are carried out.

#### 3.1.4. Resulting Model Parameters for the Burgers Model

**Figure 6:**
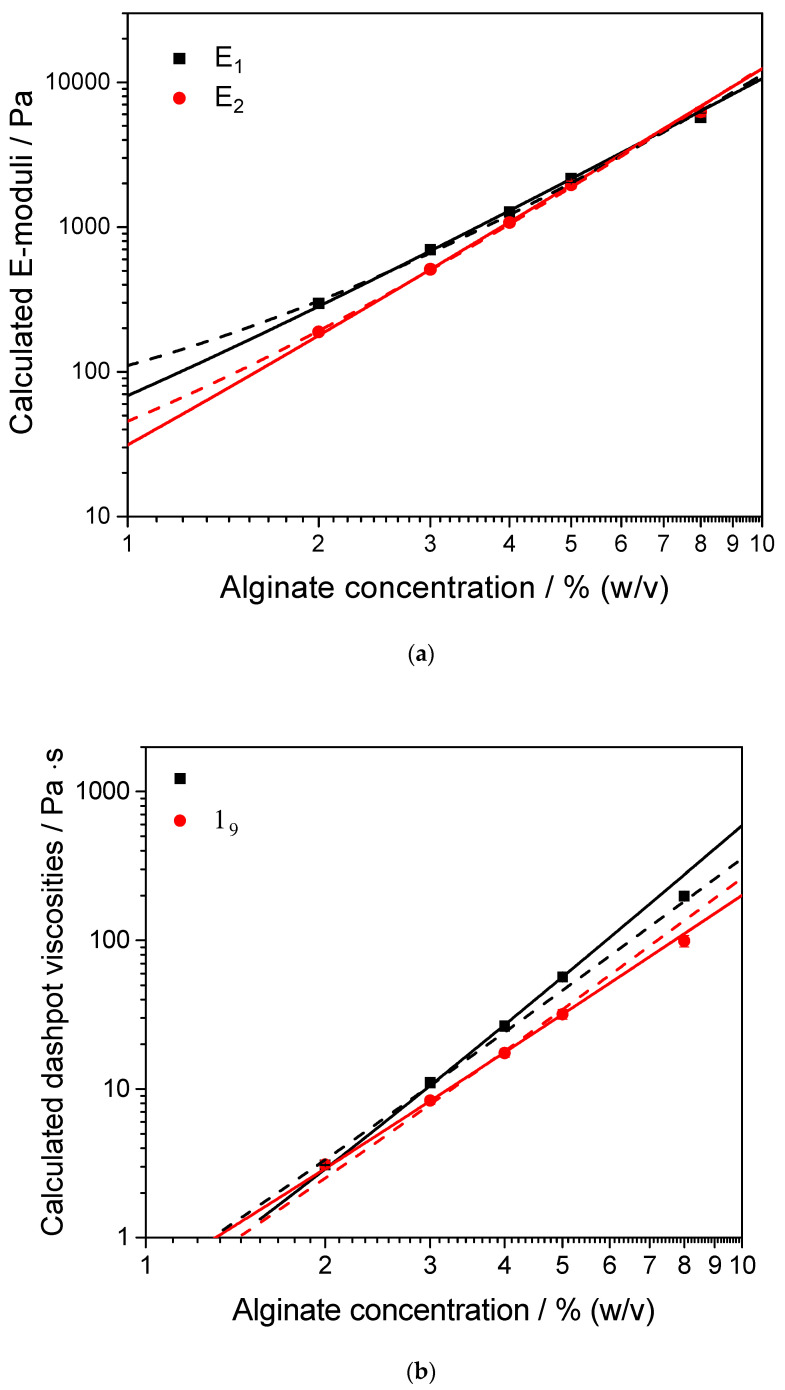
Resulting E-moduli (**a**) and Dashpot viscosities (**b**) for the Burgers model. Solid lines represent the power law like fit according to [51], dashed lines are fitted with a fixed scaling exponent of n = m = 3.

The following Figure 6 shows the calculated parameter values plotted against the alginate concentration. Error-bars are not displayed, since the calculated errors are insignificant compared to the resulting values (error bars are smaller than symbols).

By replotting the characteristic values on a double-linear, linear–log, and log–log scale their mathematical behavior becomes evident. Since a linear trend on a log–log scaled graph can be observed, a power-law-like behavior is concluded, which is also represented by the shown fits. Since non-crosslinked alginate solutions can be described as two component composites, consisting of the solvent (mainly water) and the polymer, the following quasi power-law functions can be applied for describing the determined moduli dependent on the polymer concentration [51].

(15) $$\eta \left(c\right)=\eta_{0}{\left(1+\frac{c}{\tilde{c}}\right)}^{n}$$

The zero-concentration viscosity $\eta_{0}$ is fixed at 1 mPas, which resembles the frequency independent viscosity of pure water at room temperature [52]. The characteristic concentration $\tilde{c}$ describes the concentration, where the calculated viscosity parameters become significantly larger compared to the defined zero-concentration viscosity.

The determined characteristic concentrations for the Maxwell and the Kelvin–Voigt dashpot are also used for the following fit of the E-moduli, which can be described by the following equation.

(16) $$E\left(c\right)=E_{0}{\left(1+\frac{c}{\tilde{c}}\right)}^{m}$$

Here, the zero-concentration E-modulus is used as a fitting parameter as well. This theoretically represents the model elastic modulus of the pure solvent. Since no E-modulus value is expected for pure water, an influencing artifact can be assumed. A possible influence is the surface tension of water at the edges of the geometry. Also, the onset of drying would result in a comparable influence. These phenomena can lead to an artificial elastic property for water. The resulting fitting parameters can be obtained from Table 4 provided below.

Comparing the resulting spring constant values, two learnings can be concluded. Firstly, the E_2_ parameter, which is given by the spring in the Kelvin–Voigt part of the Burgers model, becomes nearly 0 if no polymer is present. This is reasonable due to the assumption that the Kelvin–Voigt model is representative for regions with high polymer concentrations and therefore completely reversible deformation processes. A value slightly higher than 0 could be correlated to ionic interactions, which are induced by the salts that are dissolved in the DPBS puffer system. Additionally, the difference between spring constant values decreases with increasing alginate concentration, leading to a crossover in the region of 6.5 % alginate concentration.

However, a contrary trend, exhibiting similar values for low and diverging values for high alginate concentrations, can be observed for the dashpot viscosities. Considering the model system, the two dashpots can be clearly distinguished as a free dashpot and a “hindered” dashpot, which is restricted by the spring in parallel arrangement inside the Kelvin–Voigt system. This parallel arranged spring leads to a stress dissipation, while the elongation of both model components is identical, which leads to a lower calculated viscosity for the Kelvin–Voigt dashpot.

Scaling concepts in the literature suggest power law exponents for various mechanical moduli of 2 [53] and 2.25 [54] for isotropic polymeric materials. M.L. Oyen reevaluated those values [55] regarding Agarose and acrylamide hydrogels. Different mechanical moduli (Young’s modulus E, value of shear modulus |G*|, Storage modulus G’) are plotted against the total polymer concentration, revealing a power law correlation. He concluded both exponent values to be suitable for describing the analyzed material parameters. Table 4 summarizes the exponents. However, the presented data hints at slightly larger scaling exponents, since a steeper slope compared to 2 and 2.25 is clearly visible. Since our model parameter evaluation is also based on shear rheological measurements, one can assume similar correlations between model parameters and polymer content. A theoretical approach [51] reveals scaling laws with an exponent of exactly n = m = 3 for numerous isotropic material properties, such as those given in Equations (15) and (16). This approach, however, will be in the scope of future work. Nevertheless it must be pointed out that even using n = m = 3 as fixed enables excellent fits (see also Table 5) for relevant concentrations, indistinguishable from those shown in Figure 6, however, with the advantage of a reduced number of adjustable parameters for the fit procedure. Only the zero concentration viscosities remained as fixed but can be used as fitting parameter for uncharacterized solvent systems.

#### 3.1.5. Correlation of Rheological Model Parameters and Key Processing Parameters

**Figure 7:**
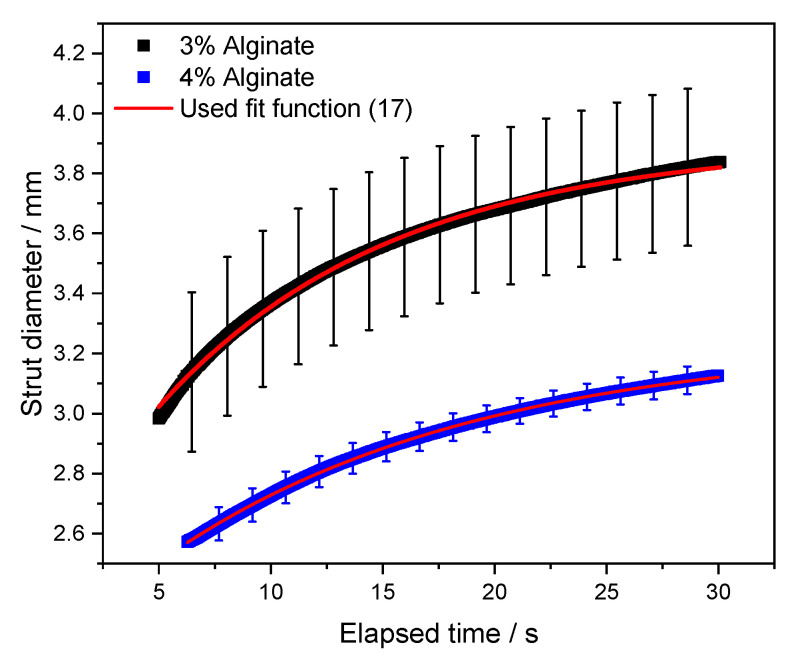
Time dependent strut diameter of 3% and 4% alginate solutions.

The initial motivation, while elaborating the presented method, is the correlation between rheological measurements and the shape fidelity of bioinks for biofabrication processes. However, characterizing the shape fidelity is anything but a trivial task. Multiple research groups published scientific articles, trying to correlate rheological material properties and the shape fidelity, often also named printability [22,56]. The usual approach for this evaluation is based on printing a single or multi layered grid. Afterwards different ratios can be calculated, e.g. resulting strut diameter divided by nozzle size [57] or a shape index including the pore geometry [58]. All these characterization techniques are based on the analysis of the strut spreading behavior. Therefore, we optically measured the strut diameter over time d(t) and fitted the resulting data using a simple exponential function, valid for the asymptotic regime (Equation (17)) [59]. Therefore, correlations to the initial strut diameter or nozzle diameter would be misleading. Figure 7 shows typical examples of the strut diameter kinetics, where 10 separately printed struts are averaged. The 3% and 4% alginate samples were printed with a constant ratio of throughput and printing speed to assure comparability.

(17) $$d\left(t\right)=A\cdot \left[1-e^{-\left(\frac{t+t_{0}}{\tau_{s}}\right)}\right]$$

d(t) = Time dependent strut diameter; A = Equilibrium strut diameter; t = Elapsed time; t_0_ = Delay time to allow a finite strut diameter at t = 0; τ_s_ = Characteristic time for spreading.

Since the measurement does not capture the (0/0) starting point, we introduced the delay time t_0_ to allow a finite strut diameter at t = 0. Furthermore, a characteristic time τ_s_ is used to quantify the spreading behavior. This characteristic time resembles the time point where the strut reaches 63.2% of the equilibrium strut diameter. Here a higher characteristic time resembles slower spreading and would therefore result in a better shape fidelity for the majority of commonly used shape fidelity assessments. The asymptotic strut diameter for infinite times can potentially be correlated to printing results as well. However, an artifact free determination of this value can be highly challenging due to several environmental effects, such as drying of hydrogel or inhomogeneity of the used printbed such as scratches, dirt particles, or a slight tilt angle. The resulting fitting parameters are shown in Table 6.

For a Burgers model, also two characteristic time constants can be calculated. One for the Maxwell part ($\tau_{1}=\frac{\eta_{1}}{E_{1}}$) and one for the Kelvin–Voigt part ($\tau_{2}=\frac{\eta_{2}}{E_{2}}$), respectively. All values for Burgers model parameters are used according to Figure 7. The results are highlighted in Table 7.

Our results indicate that τ_1_ is proportional to the characteristic spreading time τ_s_ of printed struts:

(18) $$\tau_{s}\;\sim {\;\tau }_{1}\;\sim \;\frac{\eta_{1}}{E_{1}}$$

while the proportionality factor depends on surface tension, interfacial energy and gravity [59,60].

For the investigated concentrations, un-crosslinked alginate shows the same trend for the directly assessed τ-ratio and the Maxwell-τ-ratio within the experimental error. The Kelvin–Voigt-τ-ratio does not change between 3% and 4% alginate. Therefore, it is evident from Table 7 that

(19) $$\frac{\tau_{s}\left(4\%\right)}{\tau_{s}\left(3\%\right)}\approx \frac{\tau_{1}\left(4\%\right)}{\tau_{1}\left(3\%\right)}$$

holds within the experimental error. This finding will be discussed in detail in our following publication [61]. However, one can correlate this finding to the theoretical behavior of each part of the model. As shown in “Section 3.1.2”, the Maxwell part of the Burgers model is directly correlated to the viscoelastic liquid behavior of the material and, therefore, to non-recoverable deformation, which is resembled by strut spreading in the used experimental set-up. The characteristic time of the Kelvin–Voigt model part is directly connected to the visco-elastic solid material behavior. Therefore, the characteristic time of this model part is defined by intermolecular forces and could be altered by chain-chain crosslinking. In the case of un-crosslinked alginate solutions, this can be realized by adding calcium ion, which are known to form ionic bonds between alginate molecules. Since we did not add any crosslinking agents, no change in the characteristic time of the Kelvin–Voigt-part is to be expected.

### 3.2. Rheological Model Evaluation of Further Materials

An analogous analysis of model parameters can be performed for a variety of materials. However, this would exceed the scope of this paper. Therefore, reduced analysis for Elastosil 7670, Elastollan 1180A melt and fiber filled alginate solution is presented.

#### 3.2.1. Analysis of Elastosil 7670

For determining the most suitable model system for the crosslinked silicon rubber, we compare the adjusted R^2^ parameter. This parameter describes the quality of a fit function adjusted to the number of terms used. This is especially helpful for the identification of the most simple, yet suitable rheological model system. The values, calculated by Statistica, are given in the following Table 8.

**Figure 8:**
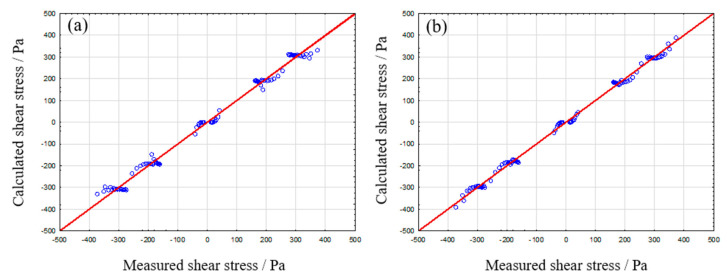
Measured shear stress against calculated shear stress for Kelvin–Voigt (**a**) and Zener m/k (**b**).

It is evident that two models are well applicable for the Elastosil sample, namely Kelvin–Voigt and Zener m/k. Both model systems resulted in an adjusted R^2^ greater than 0.99. For further analysis, a comparison of the resulting observed against predicted plots is carried out (see Figure 8). Due to the nearly fully elastic material properties of Elastosil 7670 one could already assume the Kelvin–Voigt or Zener model as suitable from a theoretical point of view. Both model systems are describing the measured data accurately.

The resulting model component parameters (see Table 9) can be calculated in a similar manner as already shown for alginate samples. The Kelvin–Voigt parameter identification reveals an overly dominant spring in parallel arrangement to a comparably low viscous dampener. This results in nearly pure elastic model behavior without rest-deformation and is therefore in good agreement with the observed material behavior of crosslinked silicon rubber systems. The same conclusions are applicable for the Zener m model. The E-moduli for the dominant springs range within an error of 5%. Additionally, calculated model systems describe elastic material behavior with no rest deformation on the tested timescale. For a final decision, whether the Zener or Kelvin–Voigt model should be used, two further considerations must be done. First, the amount of measurement data must be sufficient to permit the use of higher order model systems such as Zener or Burgers model. Otherwise, one could draw falsified conclusions, similar to fit functions while using too many parameters. Finally, the analysis of the residual distribution, which describes the difference between observed and calculated data, can yield the required information for determining the optimal model system for each material. This approach is highlighted in the following Section 3.2.2.

#### 3.2.2. Analysis of TPU 1180A

The analysis of polymer melts can potentially yield information about the macromolecular state of the polymer chains. The analysis of degradation behavior and the influence on key processing parameters in correlation to model parameters is currently performed at the Institute of Polymer Physics and Processing (Prof. Dirk W. Schubert, Erlangen, Germany) and will be further discussed in future publications. For demonstration of the applicability of our method for such materials, only a single frequency sweep will be discussed. The first step, as for all materials, is the determination of a suitable model system. Table 10 presents the initial results.

Initially, no clear decision for the best performing model system can be made. For further quantification of quality for the Zener, Lethersich/Jeffreys, and Burgers model, the residual distribution should be considered.

**Figure 9:**
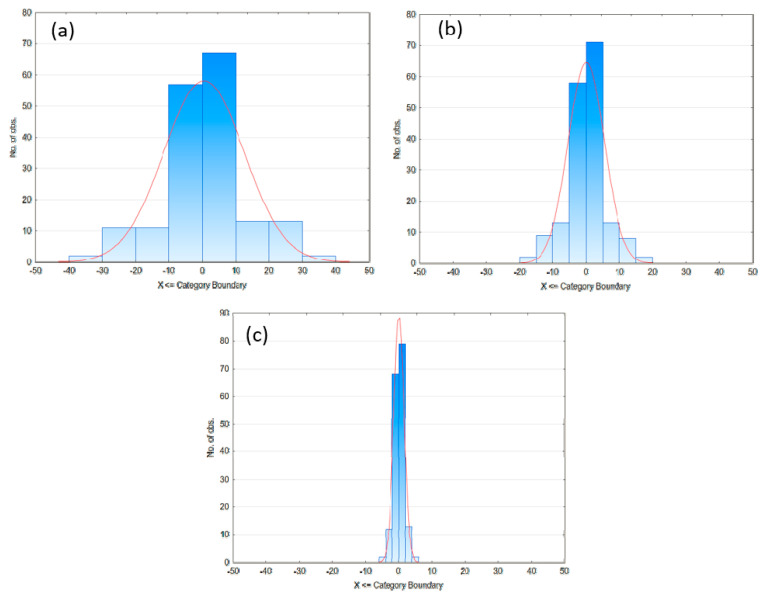
Residual distribution of Zener (**a**), Lethersich/Jeffreys (**b**), and Burgers model (**c**).

After analyzing the residual distribution (see Figure 9), it becomes evident, that the Burgers model is the most suitable model systems for the TPU 1180A polymer melt, since the narrowest distribution of residuals resembles the smallest deviations between calculated and measured data. Calculating the model parameters according to Equations (11)–(14) yields the following results, shown in Table 11.

#### 3.2.3. Analysis of PCL Filled Alginate

As a final example for the applicability of our method, we are using a 3% (*w/v*) alginate solution as in Section 3.1 with additional 10 wt % polycaprolactone fiber fragments, which are produced via electrospinning. The length of the fiber fragments varies around 10–50 μm with an average diameter of 3 μm. A detailed description is given in [61].

The Burgers model being the most suitable model system for this composite is evident (Table 12). This further enables a comparison between pure and filled alginate to evaluate the influence of fiber fragments on the rheological model parameters of the Burgers model. The results of this analysis are highlighted in Table 13.

A drastic, over 10-fold increase of η_1_ (the dashpot in serial alignment) can be observed. All other parameters are increasing by a factor 4–7 as well. Those observations can potentially be correlated to key process parameters, such as shape fidelity in biofabrication process or cell viability for biological experiments. Similar experimental results are currently investigated in the framework of the SFB TRR225 (http://trr225biofab.de/) and will surely yield new insights.

### 3.3. Theoretical Comparison to Already Existing Methods for Model Parameter Determination

Finally, as already mentioned in the introduction, one can argue that model parameters are accessible by analyzing relaxation experiments. However, this approach is only reliable for simple models like the Maxwell-model, exhibiting only two adjustable parameters. Assuming a material requiring a more complex rheological model, such as the Burgers-model, as shown above in Section 3.1, is necessary.

**Figure 10:**
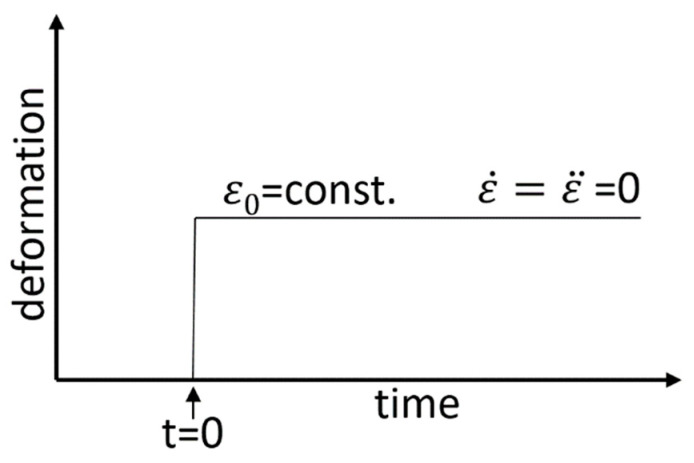
Typical relaxation experiment, applying a prompt deformation ε_0_.

A typical relaxation experiment, according to Figure 10, applies a prompt, constant deformation to $\varepsilon_{0}$ at time zero and the corresponding stress relaxation is measured.

Considering the differential equation for the Burgers model:

(20) $$\sigma +\left(\frac{E_{1}\eta_{1}+E_{1}\eta_{2}+E_{2}\eta_{1}}{E_{1}E_{2}}\right)\dot{\sigma }+\frac{\eta_{1}\eta_{2}}{E_{1}E_{2}}\ddot{\sigma }=\eta_{1}\dot{\varepsilon }+\frac{\eta_{1}\eta_{2}}{E_{2}}\ddot{\varepsilon }$$

where the right side of Equation (20) becomes zero for the scenario as shown in Figure 10 (for t > 0), thus only two pre-factors remain, yielding a solution for σ(t) of Equation (20) with two different decaying exponential functions, where the characteristic time constants are related to the remaining pre-factors. Additionally, the spring constant E_1_ is accessible from the $\varepsilon_{0}$ deformation and initial stress measured immediately after the deformation step. Therefore, a typical relaxation experiment reveals only three out of four parameters of a Burgers model, where one parameter must be chosen arbitrarily. Similar problems also occur in creep recovery experiments [17]. Nevertheless, it must be pointed out that a huge variety of data from stress relaxation, creep and creep recovery experiments or even linear increasing loading and unloading, with varying loading/unloading speeds, can enable data sets where one can generate tables like Table 1 and can perform the same statistical analysis as suggested in this work. However, only by considering rheological measurements in oscillation mode and frequency sweeps a sufficient large and reliable variation of $\varepsilon ,\dot{\varepsilon }$, $\ddot{\varepsilon },$ $\dot{\sigma },\;\ddot{\sigma }$ is accessible to model σ to reveal the material model.

Thus, our approach, presented in Section 2.1, based on oscillatory measurements, is preferable and circumvents arbitrary guesses for data interpretation.

On the other hand, one can argue from a differential equation that one can calculate the real E’ and imaginary part E″ and the corresponding frequency dependencies and fit it to the data. This seems apparently a possible approach, however, one has to fit two function at the same time otherwise one would have problems to reveal suitable model parameters. Sun et al. [15] encountered exactly this problem, where fitting of G’ data was possible with the calculated function resulting from the Burgers model, while G″ data was not. Furthermore, a fit routine requires a good guess for start values, which can be a challenge. Therefore, we reduced this problem to a linear regression and pave the way for a clear evaluation of model systems to reveal the best model under the constraint of minimal number of adjustable parameters.

## 4. Conclusions

A general, simple novel approach is presented to reveal the equations describing materials in the linear regime utilizing easily accessible data from the rheometer or DMA. The approach is applied to alginate solutions, a filled alginate solution, a crosslinked liquid silicon rubber and a TPU polymer melt. Both hydrogel samples, unfilled and fiber reinforced, could be well described by the Burgers model. Furthermore, the concentration dependent model parameters could be described by a power-law-like function with a fixed exponent of three. A subsequent study showed the correlation of hydrogel strut spreading after 3D-printing and the parameters of the Maxwell-part of the used Burgers model. The Burgers model was also well applicable for the characterized TPU melt. The LSR sample, however, was best described by the Zener model, while the Kelvin–Voigt model was also sufficiently suitable. Concluding, the presented approach can be applied to a huge variety of material classes such as polymer solutions, polymer melts, gels, or even nonwovens and composites thereof, independent of the type of deformation, shear, compression, or tensile. Potentially, the generated data can be used to correlate frequency dependent measurements with process relevant influences. In addition, a better understanding of macromolecular processes can be achieved.

## Figures and Tables

**Table 1:** Structure of the data—calculable from the rheological measurements.

ε	$\dot{\varepsilon }$	$\ddot{\varepsilon }$	σ	$\dot{\sigma }$	$\ddot{\sigma }$	ω
$\varepsilon \left(t_{11}\right)$	$\dot{\varepsilon }\left(t_{11}\right)$	$\ddot{\varepsilon }\left(t_{11}\right)$	$\sigma \left(t_{11}\right)$	$\dot{\sigma }\left(t_{11}\right)$	$\ddot{\sigma }\left(t_{11}\right)$	$\omega_{1}$
$\varepsilon \left(t_{21}\right)$	$\dot{\varepsilon }\left(t_{21}\right)$	$\ddot{\varepsilon }\left(t_{21}\right)$	$\sigma \left(t_{21}\right)$	$\dot{\sigma }\left(t_{21}\right)$	$\ddot{\sigma }\left(t_{21}\right)$	$\omega_{1}$
….	…..	…..	…..	…..	…..	….
$\varepsilon \left(t_{91}\right)$	$\dot{\varepsilon }\left(t_{91}\right)$	$\ddot{\varepsilon }\left(t_{91}\right)$	$\sigma \left(t_{91}\right)$	$\dot{\sigma }\left(t_{91}\right)$	$\ddot{\sigma }\left(t_{91}\right)$	$\omega_{1}$
$\varepsilon \left(t_{12}\right)$	$\dot{\varepsilon }\left(t_{12}\right)$	$\ddot{\varepsilon }\left(t_{12}\right)$	$\sigma \left(t_{12}\right)$	$\dot{\sigma }\left(t_{12}\right)$	$\ddot{\sigma }\left(t_{12}\right)$	$\omega_{2}$
$\varepsilon \left(t_{22}\right)$	$\dot{\varepsilon }\left(t_{22}\right)$	$\ddot{\varepsilon }\left(t_{22}\right)$	$\sigma \left(t_{22}\right)$	$\dot{\sigma }\left(t_{22}\right)$	$\ddot{\sigma }\left(t_{22}\right)$	$\omega_{2}$
….	…..	…..	…..	…..	…..	…..
$\varepsilon \left(t_{92}\right)$	$\dot{\varepsilon }\left(t_{92}\right)$	$\ddot{\varepsilon }\left(t_{92}\right)$	$\sigma \left(t_{92}\right)$	$\dot{\sigma }\left(t_{92}\right)$	$\ddot{\sigma }\left(t_{92}\right)$	$\omega_{2}$
……….	……….	……….	……….	……….	……….	……….
……….	……….	……….	……….	……….	……….	……….
……….	……….	……….	……….	……….	……….	……….
……….	……….	……….	……….	……….	……….	$\omega_{\max}$

**Table 2:** Overview of rheological models with constitutive equations of first order differential equation type, having 2 or 3 main effects Ki.

Model Name	Scheme	σ−Form of the Differential Equation	Linear Equation
Maxwell	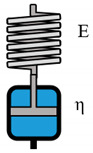	$\sigma =\eta \dot{\varepsilon }-\frac{\eta }{E}\dot{\sigma }$	$\sigma ={\;K}_{1}\dot{\varepsilon }+{\;K}_{2}\dot{\sigma }$
Kelvin-Voigt	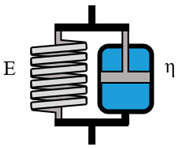	$\sigma =E\varepsilon +\eta \dot{\varepsilon }$	$\sigma =K_{1}\varepsilon +{\;K}_{2}\dot{\varepsilon }$
Zener K	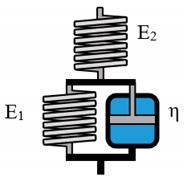	$\sigma =\frac{E_{1}E_{2}}{E_{1}+E_{2}}\varepsilon +\frac{E_{2}\eta }{E_{1}+E_{2}}\dot{\varepsilon }-\frac{\eta }{E_{1}+E_{2}}\dot{\sigma }$	$\sigma ={\;K}_{1}\varepsilon +{\;K}_{2}\dot{\varepsilon }+K_{3}\dot{\sigma }$
Zener M	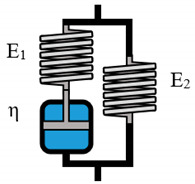	$\sigma =E_{2}\varepsilon +\frac{\eta \left(E_{1}+E_{2}\right)}{E_{1}}\dot{\varepsilon }-\frac{\eta }{E_{1}}\dot{\sigma }$	$\sigma ={\;K}_{1}\varepsilon +{\;K}_{2}\dot{\varepsilon }+K_{3}\dot{\sigma }$

**Table 3:** Overview of rheological models with constitutive equations of second order differential equation type, having 3 or 4 main effects Ki.

Model Name	Scheme	σ−Form of the Differential Equation	Linear Equation
Lethersich	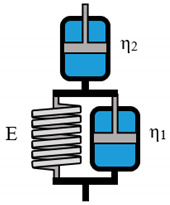	$\sigma =\eta_{2}\dot{\varepsilon }+\frac{\eta_{1}\eta_{2}}{E}\ddot{\varepsilon }-\frac{\eta_{1}+\eta_{2}}{E}\dot{\sigma }$	$\sigma =K_{1}\dot{\varepsilon }+K_{2}\ddot{\varepsilon }+K_{3}\dot{\sigma }$
Jeffreys	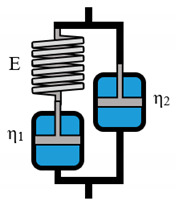	$\sigma =\left(\eta_{1}+\eta_{2}\right)\dot{\varepsilon }+\frac{\eta_{1}\eta_{2}}{E}\ddot{\varepsilon }-\frac{\eta_{1}}{E}\dot{\sigma }$	$\sigma =K_{1}\dot{\varepsilon }+K_{2}\ddot{\varepsilon }+K_{3}\dot{\sigma }$
Burgers	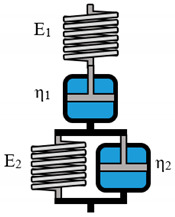	$\sigma =\eta_{1}\dot{\varepsilon }+\frac{\eta_{1}\eta_{2}}{E_{2}}\ddot{\varepsilon }-\left(\frac{E_{1}\eta_{1}+E_{1}\eta_{2}+E_{2}\eta_{1}}{E_{1}E_{2}}\right)\dot{\sigma }-\frac{\eta_{1}\eta_{2}}{E_{1}E_{2}}\ddot{\sigma }$	$\sigma =K_{1}\dot{\varepsilon }+K_{2}\ddot{\varepsilon }+K_{3}\dot{\sigma }+K_{4}\ddot{\sigma }$

**Table 4:** Resulting fit parameters for the given functions, according to Equations (15) and (16).

Parameter in Burgers Model	E_0_/η_0_ (Pa/Pa∙s)	$\tilde{c}$ (g/l)	Exponent m/n
E_1_(c)	1.18 ± 0.21	0.23 (fixed)	2.37 ± 0.06
E_2_(c)	0.06 ± 0.01	0.11 (fixed)	2.71 ± 0.03
η_1_(c)	0.001 (fixed)	0.23 ± 0.02	3.49 ± 0.08
η_2_(c)	0.001 (fixed)	0.11 ± 0.01	2.70 ± 0.04

**Table 5:** Resulting fit parameters for fixed scaling exponents, according to Equations (15) and (16).

Parameter in Burgers Model	E_0_/η_0_ (Pa/Pa∙s)	$\tilde{c}$ (g/l)	Exponent m/n
E_1_(c)	22.91 ± 5.67	1.45 ± 0.17	3
E_2_(c)	2.62 ± 0.71	0.63 ± 0.07	3
η_1_(c)	0.001 (fixed)	0.14 ± 0.01	3
η_2_(c)	0.001 (fixed)	0.16 ± 0.01	3

**Table 6:** Resulting fit parameters for the used asymptotic exponential fit function from Equation (17).

Alginate Concentration	A (mm)	t_0_ (s)	τ_s_ (s)
3%	3.90 ± 0.002	10.65 ± 0.08	10.49 ± 0.05
4%	3.24 ± 0.001	15.61 ± 0.05	13.92 ± 0.04

**Table 7:** Resulting characteristic times for 3% and 4% Alginate solutions concerning spreading and Burgers model.

	Char. Time (s)	3% Alginate	4% Alginate	τ(4%)/τ(3%)
Spreading	τ_s_	10.49 ± 0.52	13.93 ± 0.41	1.33 ± 0.01
Maxwell-Part	τ_1_	0.016 ± 0.001	0.021 ± 0.001	1.29 ± 0.137
Kelvin–Voigt-Part	τ_2_	0.017 ± 0.001	0.018 ± 0.001	1.06 ± 0.153

**Table 8:** Applicability of different rheological model systems for Elastosil 7670.

Rheological Model System	Adjusted R^2^
Maxwell	0.286
Kelvin–Voigt	0.992
Zener m/k	0.996
Lethersich/Jeffreys	0.326
Burgers	0.457

**Table 9:** Spring moduli and dashpot viscosities for Maxwell, Kelvin–Voigt, and Zener m model.

Rheological Model System	E_1_	η	E_2_
	(Pa)	(Pa∙s)	(Pa)
Kelvin–Voigt	32,685.11	54.18	/
Zener m	6926.5	138.2	31,340.72

**Table 10:** Applicability of different model systems for the thermoplastic poly-urethane (TPU) 1180A melt.

Rheological Model System	Adjusted R^2^
Maxwell	0.997
Kelvin–Voigt	0.960
Zener m/k	0.997
Lethersich/Jeffreys	0.999
Burgers	0.999

**Table 11:** Calculated Burgers parameter for Elastollan 1180A, according to Equations (11)–(14).

Model Parameter in Burgers Model	Elastollan 1180A
E_1_ (Pa)	203,959.30 ± 5765.51
E_2_ (Pa)	116,297.58 ± 7.06
η_1_ (Pa∙s)	403.75 ± 0.48
η_2_ (Pa∙s)	1662.26 ± 27.00

**Table 12:** Applicability of different model systems for PCL filled 3% (*w/v*) alginate solution.

Rheological Model System	Adjusted R^2^
Maxwell	0.828
Kelvin–Voigt	0.639
Zener m/k	0.924
Lethersich/Jeffreys	0.859
Burgers	0.952

**Table 13:** Influence of 10% PCL fiber fragments on the Burgers model parameters of 3% (*w/v*) alginate.

Model Parameter	Pure 3% Alginate	3% Alginate + 10 wt % PCL	Percentage Increase
E_1_ (Pa)	697.69 ± 35.38	2916.71 ± 215.47	418%
E_2_ (Pa)	511.27 ± 22.54	2611.20 ± 7.06	511%
η_1_ (Pa∙s)	11.07 ± 0.12	139.74 ± 4.23	1262%
η_2_ (Pa∙s)	8.38 ± 0.47	54.50 ± 3.26	650%
